# 4,4′-Dimeth­oxy-2,2′-{[(3a*RS*,7a*RS*)-2,3,3a,4,5,6,7,7a-octa­hydro-1*H*-1,3-benzimidazole-1,3-diyl]bis(methyl­ene)}diphenol

**DOI:** 10.1107/S1600536811031436

**Published:** 2011-08-11

**Authors:** Augusto Rivera, Diego Quiroga, Jaime Ríos-Motta, Karla Fejfarová, Michal Dušek

**Affiliations:** a Departamento de Química, Universidad Nacional de Colombia, Ciudad Universitaria, Bogotá, Colombia; b Institute of Physics ASCR, v.v.i., Na Slovance 2, 182 21 Praha 8, Czech Republic

## Abstract

The title compound, C_23_H_30_N_2_O_4_, is a Mannich base useful for studying the effect of an electron-donating phenol substituent on intra­molecular hydrogen bonding. In the mol­ecular structure, the cyclo­hexane ring adopts a chair conformation and the five-membered ring has a twisted envelope conformation. Each meth­oxy group is oriented in the same plane of the respective aromatic ring, showing torsion angles below 11.8 (3)° and bond angles between the meth­oxy group and the aromatic ring of 116.6 (2) and 116.6 (1)°. The structure shows inter­actions between two the N atoms of the heterocyclic ring and the hy­droxy groups by intra­molecular O—H⋯N hydrogen-bonding inter­actions. In the crystal, C—H⋯O inter­actions are observed. The crystal studied was a racemic mixture of *RR* and *SS* enanti­omers.

## Related literature

For related structures, see: Rivera *et al.* (2010*a* ▶,*b* ▶). For the effect of the meth­oxy group on mol­ecular structure, see: Özek *et al.* (2008 ▶); Ünver *et al.* (2009 ▶); Jamjah *et al.* (2011 ▶). For related quantum-chemical literature, see: Konschin (1984 ▶).
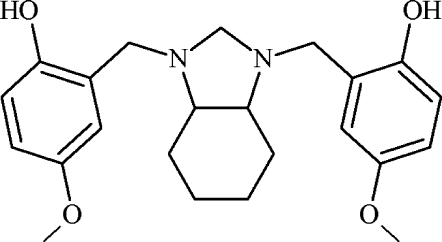


## Experimental

### 

#### Crystal data

C_23_H_30_N_2_O_4_
*M* *_r_* = 398.5
Monoclinic, 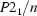

*a* = 12.7693 (3) Å
*b* = 10.4365 (2) Å
*c* = 16.3229 (4) Å
β = 109.579 (3)°
*V* = 2049.53 (9) Å^3^
*Z* = 4
Cu *K*α radiation
μ = 0.71 mm^−1^
*T* = 120 K
0.51 × 0.14 × 0.02 mm

#### Data collection

Agilent Xcalibur diffractometer with an Atlas (Gemini ultra Cu) detector
Absorption correction: multi-scan (*CrysAlis PRO*; Agilent, 2010 ▶) *T*_min_ = 0.403, *T*_max_ = 1
23926 measured reflections
3216 independent reflections
2577 reflections with *I* > 3σ(*I*)
*R*_int_ = 0.055

#### Refinement

*R*[*F* ^2^ > 2σ(*F* ^2^)] = 0.037
*wR*(*F* ^2^) = 0.105
*S* = 1.70
3216 reflections
268 parameters
2 restraints
H atoms treated by a mixture of independent and constrained refinement
Δρ_max_ = 0.19 e Å^−3^
Δρ_min_ = −0.17 e Å^−3^

### 

Data collection: *CrysAlis PRO* (Agilent, 2010 ▶); cell refinement: *CrysAlis PRO*; data reduction: *CrysAlis PRO*; program(s) used to solve structure: *SIR2002* (Burla *et al.*, 2003 ▶); program(s) used to refine structure: *JANA2006* (Petříček *et al.* 2006 ▶); molecular graphics: *DIAMOND* (Brandenburg & Putz, 2005 ▶); software used to prepare material for publication: *JANA2006*.

## Supplementary Material

Crystal structure: contains datablock(s) global, I. DOI: 10.1107/S1600536811031436/nr2009sup1.cif

Structure factors: contains datablock(s) I. DOI: 10.1107/S1600536811031436/nr2009Isup2.hkl

Supplementary material file. DOI: 10.1107/S1600536811031436/nr2009Isup3.cml

Additional supplementary materials:  crystallographic information; 3D view; checkCIF report

## Figures and Tables

**Table 1:** Hydrogen-bond geometry (Å, °)

*D*—H⋯*A*	*D*—H	H⋯*A*	*D*⋯*A*	*D*—H⋯*A*
O1—H1⋯N2	0.89 (2)	1.90 (2)	2.709 (2)	151.1 (19)
O3—H3⋯N1	0.88 (2)	1.91 (2)	2.706 (2)	150.0 (19)
C8—H8A⋯O2^i^	0.96	2.55	3.427 (2)	152

Symmetry code: (i) 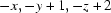.

**Fig. 1.**
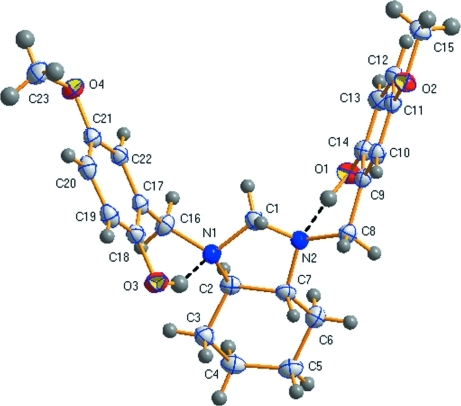
A view of (I) with the numbering scheme. Displacement ellipsoids are drawn at the 50% probability level. Dashed lines indicate intramolecular hydrogen bonds.

**Fig. 2.**
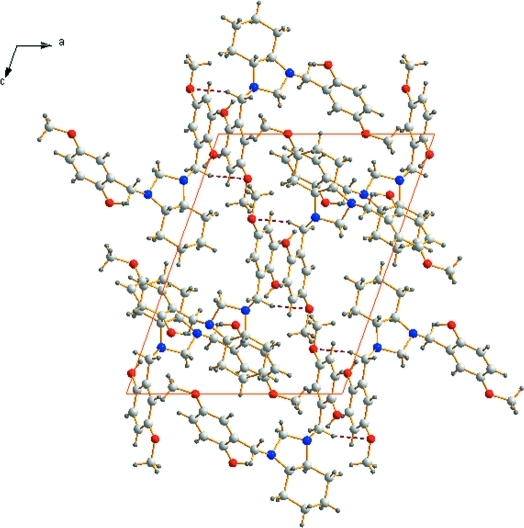
Packing of the molecules of the title compound. Dashed lines indicate weak intermolecular hydrogen bonds.

## supplementary crystallographic information

### Comment

During our investigations on Mannich bases, we studied the effect of electron-withdrawing or electron-donating substituent of phenol on intramolecular hydrogen bond. Here we report the structure of the title compound (Fig. 1). The X-ray results of the title compound suggest an influence of the methoxy substituent in the hydrogen bonding interaction. The N···H distances and the N···O distances are longer (by about 0.07 Å and 0.05 Å, respectively), than the observed values in a related structure where the *p*-substituent is a chlorine atom (Rivera, *et al.* 2010*b*). However these values are in good agreement with the one found in the related structure where there are not *p*-substituents [N···H, 1.91 (2) Å; N···O, 2.6894 (14) Å] (Rivera *et al.*, 2010*a*). Moreover, the observed C—O bond lengths [C10—O1, 1.377 (2) Å; C18—O3, 1.373 (2) Å] are longer in relation to the mentioned related structures [C—O, 1.364 (2) Å, C—O, 1.354 (2) Å] (Rivera *et al.*, 2010**a*,b*), which confirms the decreasing in the intermolecular hydrogen bonding interaction due the electronic influence of a electron-donating substituent as the methoxy group. The crystal packing (Figure 2) displays weak intermolecular C—H···O hydrogen bonds between neighboring molecules, which link them into 1D-chains.

In the crystal structure of the title compound, the cyclohexanediamine fragment adopts a chair conformation. The C—C—C bond angles within the cyclohexane ring are close to normal tetrahedral bond angles in a chair conformation since these values are in the range of 108.4 (2)° to 112.8 (2)°. The imidazolidine moiety has a twisted envelope conformation, indicating that the nitrogen lone pairs are oriented anti-axial to avoid repulsion electronic repulsions. In comparison with the values of the corresponding angles and bond distances in the phenol derivative (Rivera *et al.*, 2010*a*), the C12—C13—O2 and C20—C21—O4 angles increase by 3.48° and 4.27° respectively, and the C9—C10 and C17—C18 bonds are the longest and the C10—C11 and C18—C19 bond are the shortest in the aromatic rings. These results suggest the existence of a distortion in the aromatic rings, which is present at the *p*-methoxyphenol moiety in some Schiff bases (Özek *et al.*, 2008; Ünver *et al.*, 2009; Jamjah *et al.*, 2011), which could be explained by the presence of the OH and CH_3_ groups, such as an STO-3G molecular orbital investigation suggested (Konschin, 1984), where molecular structure optimizations of methoxy-containing benzenes and related compounds indicated significant structural consequences in the aromatic rings by the presence of these substituents due their behavior as π donors and σ acceptors.

### Experimental

To a solution of (2R,7R,11S,16S)-1,8,10,17-tetraazapentacyclo- [8.8.1.1^8,17^.0^2,7^.0^11,16^]icosane (276 mg, 1.00 mmol) in dioxane (3 mL) and water (4 mL) in a two-necked round-bottomed flask, prepared beforehand following previously described procedures, was added dropwise a dioxane solution (3 mL) containing two equivalents of 4-methoxyphenol (248 mg, 2.00 mmol). The mixture was refluxed for about 8h. The solvent was evaporated under reduced pressure until a sticky residue appeared. The product was purified by chromatography on a silica column, and subjected to gradient elution with benzene:ethyl acetate (yield 34%, m.p. = 436–438 K). Single crystals of titlt compound were grown from a chloroform: methanol solution by slow evaporation of the solvent at room temperature over a period of about 2 weeks.

### Refinement

The hydrogen attached to C atoms were positioned geometrically and kept in ideal positions with C–H distance 0.96 Å during the refinement. The hydroxyl H atoms were found in difference Fourier maps and refined with a distance restraint d(O—H) = 0.84 (2) Å. The isotropic atomic displacement parameters of hydrogen atoms set to 1.5×*U*_eq_(C,O) for methyl and hydroxyl groups and 1.2×*U*_eq_(C) for all other hydrogen atoms.

### Crystal data

**Table:**

C_23_H_30_N_2_O_4_	*F*(000) = 856
*M**_r_* = 398.5	*D*_x_ = 1.291 Mg m^−^^3^
Monoclinic, *P*2_1_/*n*	Cu *K*α radiation, λ = 1.5418 Å
Hall symbol: -P 2yn	Cell parameters from 11032 reflections
*a* = 12.7693 (3) Å	θ = 3.7–62.6°
*b* = 10.4365 (2) Å	µ = 0.71 mm^−^^1^
*c* = 16.3229 (4) Å	*T* = 120 K
β = 109.579 (3)°	Plate, colourless
*V* = 2049.53 (9) Å^3^	0.51 × 0.14 × 0.02 mm
*Z* = 4	

### Data collection

**Table:**

Agilent Xcalibur diffractometer with an Atlas (Gemini ultra Cu) detector	3216 independent reflections
Radiation source: Enhance Ultra (Cu) X-ray Source	2577 reflections with *I* > 3σ(*I*)
mirror	*R*_int_ = 0.055
Detector resolution: 10.3784 pixels mm^-1^	θ_max_ = 62.7°, θ_min_ = 3.8°
Rotation method data acquisition using ω scans	*h* = −14→14
Absorption correction: multi-scan (*CrysAlis PRO*; Agilent, 2010)	*k* = −11→11
*T*_min_ = 0.403, *T*_max_ = 1	*l* = −18→18
23926 measured reflections	

### Refinement

**Table:**

Refinement on *F*^2^	114 constraints
*R*[*F*^2^ > 2σ(*F*^2^)] = 0.037	H atoms treated by a mixture of independent and constrained refinement
*wR*(*F*^2^) = 0.105	Weighting scheme based on measured s.u.'s *w* = 1/[σ^2^(*I*) + 0.0016*I*^2^]
*S* = 1.70	(Δ/σ)_max_ = 0.003
3216 reflections	Δρ_max_ = 0.19 e Å^−^^3^
268 parameters	Δρ_min_ = −0.17 e Å^−^^3^
2 restraints	

### Special details

**Table:**

Experimental. CrysAlisPro (Agilent Technologies, 2010), empirical absorption correction using spherical harmonics, implemented in SCALE3 ABSPACK scaling algorithm. ^1^H NMR (CDCl3, 400 MHz): δ 1.28 (4H, m), 1.84 (2H, m), 2.05 (2H, m), 2.32 (2H, m), 3.40 (2H, d,J = 14.0 Hz, ArCH2N), 3.53 (2H, s, NCH2N), 3.71 (2H, s, ArOCH3), 4.16 (2H, d, J = 14.0 Hz, ArCH2N), 6.51 (2H, d, J= 2.0 Hz), 6.70 (2H, d, J = 8.8 Hz), 6.73 (2H, d, J = 8.8 Hz), 10.05 (2H, bs, ArOH). 13C NMR (CDCl3 , 100 MHz): δ 24.0, 28.9, 55.7, 56.4, 69.1, 75.8, 113.8, 113.9, 116.5, 122.1, 151.1, 152.5.
Refinement. The refinement was carried out against all reflections. The conventional *R*-factor is always based on *F*. The goodness of fit as well as the weighted *R*-factor are based on *F* and *F*^2^ for refinement carried out on *F* and *F*^2^, respectively. The threshold expression is used only for calculating *R*-factors *etc*. and it is not relevant to the choice of reflections for refinement.The program used for refinement, Jana2006, uses the weighting scheme based on the experimental expectations, see _refine_ls_weighting_details, that does not force *S* to be one. Therefore the values of *S* are usually larger than the ones from the *SHELX* program.

### Fractional atomic coordinates and isotropic or equivalent isotropic displacement parameters (Å^2^)

**Table:**

	*x*	*y*	*z*	*U*_iso_*/*U*_eq_	
O1	−0.00977 (10)	0.13997 (12)	0.92026 (9)	0.0335 (5)	
O2	0.20745 (10)	0.49247 (11)	1.17311 (8)	0.0319 (4)	
O3	0.39277 (11)	0.34923 (12)	0.73545 (9)	0.0364 (5)	
O4	0.68016 (10)	0.06056 (12)	0.99982 (8)	0.0344 (5)	
N1	0.22853 (11)	0.21558 (13)	0.76675 (9)	0.0263 (5)	
N2	0.08623 (12)	0.27229 (13)	0.82138 (9)	0.0254 (5)	
C1	0.20785 (14)	0.26197 (17)	0.84535 (12)	0.0293 (6)	
C2	0.11931 (14)	0.17745 (16)	0.70585 (11)	0.0259 (6)	
C3	0.10493 (15)	0.17697 (17)	0.61017 (12)	0.0320 (7)	
C4	−0.01711 (15)	0.14831 (17)	0.55879 (12)	0.0338 (7)	
C5	−0.09725 (15)	0.23760 (17)	0.58246 (12)	0.0321 (7)	
C6	−0.07651 (15)	0.24213 (16)	0.68027 (12)	0.0302 (7)	
C7	0.04455 (14)	0.27539 (16)	0.72600 (11)	0.0251 (6)	
C8	0.05134 (14)	0.37784 (16)	0.86593 (11)	0.0281 (6)	
C9	0.07705 (13)	0.34676 (15)	0.96068 (12)	0.0252 (6)	
C10	0.04316 (13)	0.22793 (16)	0.98343 (12)	0.0263 (6)	
C11	0.06181 (14)	0.19843 (17)	1.06953 (12)	0.0309 (7)	
C12	0.11433 (14)	0.28558 (16)	1.13485 (13)	0.0295 (6)	
C13	0.15068 (13)	0.40195 (16)	1.11302 (12)	0.0265 (6)	
C14	0.13145 (13)	0.43191 (16)	1.02638 (11)	0.0262 (6)	
C15	0.22791 (16)	0.46243 (19)	1.26223 (12)	0.0359 (7)	
C16	0.31306 (13)	0.11383 (16)	0.78596 (12)	0.0293 (6)	
C17	0.42842 (14)	0.16482 (15)	0.83063 (11)	0.0254 (6)	
C18	0.46432 (15)	0.27800 (16)	0.80128 (12)	0.0284 (6)	
C19	0.57287 (15)	0.31821 (17)	0.83832 (12)	0.0315 (7)	
C20	0.64851 (15)	0.24705 (16)	0.90374 (12)	0.0311 (7)	
C21	0.61355 (14)	0.13648 (16)	0.93405 (12)	0.0281 (6)	
C22	0.50375 (14)	0.09762 (16)	0.89813 (11)	0.0264 (6)	
C23	0.79664 (15)	0.0851 (2)	1.02718 (14)	0.0417 (8)	
H1a	0.235321	0.200329	0.89123	0.0352*	
H1b	0.240737	0.345116	0.860697	0.0352*	
H2	0.104504	0.089121	0.714225	0.0311*	
H3a	0.151109	0.111591	0.598758	0.0384*	
H3b	0.124388	0.259562	0.593708	0.0384*	
H4a	−0.029043	0.154845	0.497662	0.0405*	
H4b	−0.033578	0.061082	0.568652	0.0405*	
H5a	−0.172313	0.210762	0.552602	0.0385*	
H5b	−0.091166	0.322414	0.561738	0.0385*	
H6a	−0.122704	0.306865	0.692378	0.0362*	
H6b	−0.092114	0.159704	0.699614	0.0362*	
H7	0.04643	0.361176	0.705313	0.0301*	
H8a	−0.027093	0.391828	0.839302	0.0337*	
H8b	0.089623	0.454824	0.860471	0.0337*	
H11	0.038319	0.116981	1.084521	0.0371*	
H12	0.125363	0.265437	1.194548	0.0354*	
H14	0.156196	0.512848	1.011693	0.0314*	
H15a	0.269655	0.530503	1.298034	0.0539*	
H15b	0.158409	0.452464	1.272194	0.0539*	
H15c	0.269456	0.384083	1.276543	0.0539*	
H16a	0.3102	0.071913	0.732899	0.0352*	
H16b	0.296188	0.049866	0.821931	0.0352*	
H19	0.596496	0.396257	0.818713	0.0378*	
H20	0.724523	0.274297	0.927787	0.0373*	
H22	0.479374	0.022481	0.920516	0.0317*	
H23a	0.835156	0.023317	1.070319	0.0625*	
H23b	0.821887	0.078894	0.978153	0.0625*	
H23c	0.811304	0.169675	1.051497	0.0625*	
H1	0.0129 (19)	0.160 (2)	0.8762 (13)	0.0503*	
H3	0.3253 (14)	0.326 (2)	0.7320 (16)	0.0547*	

### Atomic displacement parameters (Å^2^)

**Table:**

	*U*^11^	*U*^22^	*U*^33^	*U*^12^	*U*^13^	*U*^23^
O1	0.0384 (7)	0.0312 (7)	0.0300 (8)	−0.0083 (5)	0.0101 (6)	−0.0029 (5)
O2	0.0367 (7)	0.0311 (7)	0.0269 (7)	−0.0039 (5)	0.0095 (6)	−0.0047 (5)
O3	0.0392 (7)	0.0326 (7)	0.0368 (8)	0.0016 (6)	0.0119 (7)	0.0087 (6)
O4	0.0267 (6)	0.0360 (7)	0.0362 (8)	0.0021 (5)	0.0049 (6)	0.0024 (5)
N1	0.0278 (7)	0.0249 (7)	0.0257 (9)	0.0024 (6)	0.0081 (7)	−0.0016 (6)
N2	0.0281 (7)	0.0249 (7)	0.0230 (8)	0.0025 (6)	0.0083 (7)	−0.0009 (6)
C1	0.0301 (9)	0.0305 (10)	0.0273 (11)	0.0008 (7)	0.0095 (8)	−0.0027 (7)
C2	0.0279 (9)	0.0235 (9)	0.0254 (10)	−0.0018 (7)	0.0079 (8)	0.0000 (7)
C3	0.0355 (10)	0.0329 (10)	0.0285 (11)	0.0008 (7)	0.0118 (9)	−0.0011 (8)
C4	0.0397 (10)	0.0321 (10)	0.0272 (11)	−0.0055 (8)	0.0081 (9)	−0.0006 (8)
C5	0.0302 (10)	0.0315 (10)	0.0298 (11)	−0.0055 (7)	0.0037 (9)	0.0005 (8)
C6	0.0289 (9)	0.0276 (9)	0.0333 (11)	−0.0005 (7)	0.0095 (8)	−0.0014 (7)
C7	0.0299 (9)	0.0214 (8)	0.0234 (10)	−0.0002 (7)	0.0082 (8)	0.0028 (7)
C8	0.0331 (9)	0.0238 (9)	0.0282 (10)	0.0043 (7)	0.0113 (8)	0.0009 (7)
C9	0.0246 (8)	0.0240 (9)	0.0286 (10)	0.0048 (7)	0.0108 (8)	0.0006 (7)
C10	0.0248 (9)	0.0255 (9)	0.0285 (11)	−0.0017 (7)	0.0086 (8)	−0.0013 (7)
C11	0.0313 (9)	0.0281 (9)	0.0343 (11)	−0.0031 (7)	0.0122 (9)	0.0034 (8)
C12	0.0284 (9)	0.0329 (10)	0.0279 (11)	0.0010 (7)	0.0102 (8)	0.0024 (8)
C13	0.0241 (8)	0.0286 (9)	0.0272 (10)	0.0030 (7)	0.0091 (8)	−0.0039 (7)
C14	0.0274 (9)	0.0210 (8)	0.0324 (11)	0.0024 (7)	0.0130 (8)	−0.0003 (7)
C15	0.0403 (11)	0.0391 (11)	0.0259 (11)	0.0020 (8)	0.0080 (9)	−0.0047 (8)
C16	0.0288 (9)	0.0246 (9)	0.0335 (11)	0.0030 (7)	0.0091 (8)	−0.0011 (7)
C17	0.0286 (9)	0.0237 (9)	0.0263 (10)	0.0016 (7)	0.0125 (8)	−0.0043 (7)
C18	0.0349 (9)	0.0259 (9)	0.0268 (10)	0.0033 (7)	0.0137 (8)	−0.0001 (7)
C19	0.0370 (10)	0.0270 (9)	0.0358 (11)	−0.0027 (7)	0.0190 (9)	−0.0039 (8)
C20	0.0294 (9)	0.0327 (10)	0.0337 (11)	−0.0038 (7)	0.0138 (9)	−0.0081 (8)
C21	0.0301 (9)	0.0286 (9)	0.0264 (10)	0.0029 (7)	0.0106 (8)	−0.0037 (7)
C22	0.0305 (9)	0.0231 (9)	0.0277 (10)	0.0013 (7)	0.0124 (8)	−0.0012 (7)
C23	0.0282 (9)	0.0524 (13)	0.0409 (13)	0.0044 (9)	0.0071 (9)	−0.0009 (10)

### Geometric parameters (Å, °)

**Table:**

O1—C10	1.377 (2)	C7—H7	0.96
O1—H1	0.89 (2)	C8—C9	1.505 (3)
O2—C13	1.3797 (19)	C8—H8a	0.96
O2—C15	1.424 (2)	C8—H8b	0.96
O3—C18	1.373 (2)	C9—C10	1.404 (2)
O3—H3	0.88 (2)	C9—C14	1.387 (2)
O4—C21	1.375 (2)	C10—C11	1.379 (3)
O4—C23	1.426 (2)	C11—C12	1.391 (2)
N1—C1	1.476 (3)	C11—H11	0.96
N1—C2	1.4705 (19)	C12—C13	1.389 (3)
N1—C16	1.471 (2)	C12—H12	0.96
N2—C1	1.473 (2)	C13—C14	1.388 (3)
N2—C7	1.467 (2)	C14—H14	0.96
N2—C8	1.469 (2)	C15—H15a	0.96
C1—H1a	0.96	C15—H15b	0.96
C1—H1b	0.96	C15—H15c	0.96
C2—C3	1.510 (3)	C16—C17	1.505 (2)
C2—C7	1.508 (3)	C16—H16a	0.96
C2—H2	0.96	C16—H16b	0.96
C3—C4	1.531 (2)	C17—C18	1.408 (3)
C3—H3a	0.96	C17—C22	1.386 (2)
C3—H3b	0.96	C18—C19	1.379 (2)
C4—C5	1.526 (3)	C19—C20	1.391 (2)
C4—H4a	0.96	C19—H19	0.96
C4—H4b	0.96	C20—C21	1.387 (3)
C5—C6	1.529 (3)	C20—H20	0.96
C5—H5a	0.96	C21—C22	1.387 (2)
C5—H5b	0.96	C22—H22	0.96
C6—C7	1.515 (2)	C23—H23a	0.96
C6—H6a	0.96	C23—H23b	0.96
C6—H6b	0.96	C23—H23c	0.96
			
C10—O1—H1	103.8 (13)	C9—C8—H8b	109.4712
C13—O2—C15	116.64 (14)	H8a—C8—H8b	108.4897
C18—O3—H3	106.3 (15)	C8—C9—C10	118.81 (14)
C21—O4—C23	116.64 (15)	C8—C9—C14	122.47 (15)
C1—N1—C2	105.73 (14)	C10—C9—C14	118.72 (17)
C1—N1—C16	112.57 (13)	O1—C10—C9	120.55 (16)
C2—N1—C16	114.24 (13)	O1—C10—C11	119.22 (15)
C1—N2—C7	105.00 (15)	C9—C10—C11	120.23 (15)
C1—N2—C8	113.02 (13)	C10—C11—C12	120.62 (17)
C7—N2—C8	116.51 (12)	C10—C11—H11	119.6883
N1—C1—N2	105.84 (13)	C12—C11—H11	119.6884
N1—C1—H1a	109.4714	C11—C12—C13	119.48 (18)
N1—C1—H1b	109.4707	C11—C12—H12	120.2628
N2—C1—H1a	109.4711	C13—C12—H12	120.2617
N2—C1—H1b	109.4719	O2—C13—C12	123.89 (16)
H1a—C1—H1b	112.872	O2—C13—C14	116.21 (15)
N1—C2—C3	117.33 (16)	C12—C13—C14	119.89 (15)
N1—C2—C7	101.27 (13)	C9—C14—C13	121.03 (16)
N1—C2—H2	110.5517	C9—C14—H14	119.4846
C3—C2—C7	111.18 (13)	C13—C14—H14	119.4868
C3—C2—H2	100.5211	O2—C15—H15a	109.4711
C7—C2—H2	116.7735	O2—C15—H15b	109.4712
C2—C3—C4	108.40 (17)	O2—C15—H15c	109.4713
C2—C3—H3a	109.471	H15a—C15—H15b	109.4715
C2—C3—H3b	109.4714	H15a—C15—H15c	109.4714
C4—C3—H3a	109.471	H15b—C15—H15c	109.4709
C4—C3—H3b	109.4714	N1—C16—C17	112.16 (13)
H3a—C3—H3b	110.5178	N1—C16—H16a	109.4712
C3—C4—C5	112.81 (15)	N1—C16—H16b	109.4704
C3—C4—H4a	109.4711	C17—C16—H16a	109.4719
C3—C4—H4b	109.4712	C17—C16—H16b	109.4714
C5—C4—H4a	109.4716	H16a—C16—H16b	106.6446
C5—C4—H4b	109.4708	C16—C17—C18	120.62 (14)
H4a—C4—H4b	105.9139	C16—C17—C22	120.81 (15)
C4—C5—C6	112.76 (14)	C18—C17—C22	118.47 (15)
C4—C5—H5a	109.4712	O3—C18—C17	120.90 (15)
C4—C5—H5b	109.471	O3—C18—C19	119.09 (16)
C6—C5—H5a	109.472	C17—C18—C19	120.02 (15)
C6—C5—H5b	109.4709	C18—C19—C20	120.78 (17)
H5a—C5—H5b	105.9654	C18—C19—H19	119.6089
C5—C6—C7	108.22 (17)	C20—C19—H19	119.6102
C5—C6—H6a	109.4719	C19—C20—C21	119.63 (16)
C5—C6—H6b	109.4716	C19—C20—H20	120.1833
C7—C6—H6a	109.4709	C21—C20—H20	120.1828
C7—C6—H6b	109.4705	O4—C21—C20	124.67 (15)
H6a—C6—H6b	110.6899	O4—C21—C22	115.77 (16)
N2—C7—C2	100.68 (12)	C20—C21—C22	119.55 (15)
N2—C7—C6	117.73 (17)	C17—C22—C21	121.48 (17)
N2—C7—H7	110.5362	C17—C22—H22	119.2602
C2—C7—C6	110.69 (14)	C21—C22—H22	119.2596
C2—C7—H7	117.589	O4—C23—H23a	109.4708
C6—C7—H7	100.4929	O4—C23—H23b	109.4712
N2—C8—C9	110.44 (13)	O4—C23—H23c	109.4711
N2—C8—H8a	109.4713	H23a—C23—H23b	109.4715
N2—C8—H8b	109.4709	H23a—C23—H23c	109.4708
C9—C8—H8a	109.471	H23b—C23—H23c	109.4719
			
C15—O2—C13—C12	−0.5 (3)	C1—N1—C16—C17	−72.95 (18)
C15—O2—C13—C14	−179.80 (16)	C2—N1—C16—C17	166.44 (14)
C23—O4—C21—C20	−11.8 (3)	C7—N2—C1—N1	−18.92 (16)
C23—O4—C21—C22	169.32 (16)	C8—N2—C1—N1	−146.94 (13)
C2—N1—C1—N2	−10.49 (16)	C1—N2—C7—C2	39.95 (16)
C16—N1—C1—N2	−135.87 (14)	C1—N2—C7—C6	160.30 (14)
C1—N1—C2—C3	155.91 (15)	C8—N2—C7—C2	165.82 (14)
C1—N1—C2—C7	34.74 (16)	C8—N2—C7—C6	−73.83 (19)
C16—N1—C2—C3	−79.74 (18)	C1—N2—C8—C9	−71.56 (18)
C16—N1—C2—C7	159.08 (14)	C7—N2—C8—C9	166.70 (15)

### Hydrogen-bond geometry (Å, °)

**Table:**

*D*—H···*A*	*D*—H	H···*A*	*D*···*A*	*D*—H···*A*
O1—H1···N2	0.89 (2)	1.90 (2)	2.709 (2)	151.1 (19)
O3—H3···N1	0.88 (2)	1.91 (2)	2.706 (2)	150.0 (19)
C8—H8A···O2^i^	0.96	2.55	3.427 (2)	152

Symmetry codes: (i) −*x*, −*y*+1, −*z*+2.
